# Genetic Polymorphism and Gene Expression of β-Defensin-1 in Periodontitis Associated With Type 2 Diabetes Mellitus

**DOI:** 10.7759/cureus.49814

**Published:** 2023-12-01

**Authors:** Harini Venkata Subbiah, Polani Ramesh Babu, Usha Subbiah

**Affiliations:** 1 Human Genetics Research Centre, Sree Balaji Dental College & Hospital, Bharath Institute of Higher Education and Research, Chennai, IND; 2 Center for Materials Engineering and Regenerative Medicine, Bharath Institute of Higher Education and Research, Chennai, IND

**Keywords:** gene expression, polymorphism, t2dm, periodontitis, defensin

## Abstract

Introduction

Periodontitis is a multifactorial disease caused by periodontopathic bacteria and influenced by both genetic and environmental factors. Genetic predispositions are found to play a crucial part in the onset and progression of periodontal disease. There is a two-way relationship between diabetes and periodontitis with severe periodontal tissue destruction seen in diabetic patients. Antimicrobial peptide, β-defensin-1 (*DEFB1* gene), plays an important role in the innate immune responses and forms the first line of host defense against periodontal pathogens. Single nucleotide polymorphisms (SNPs) in the specific genetic loci of the *DEFB1* gene and its expression level could confer a degree of risk or protection from periodontitis associated with diabetes. The present study determined the association between SNPs at the 5’ untranslated region (UTR) in the *DEFB1 *gene and susceptibility to periodontitis associated with type 2 diabetes mellitus (T2DM) and analyzed the effect of 5’ UTR polymorphisms on *DEFB1 *gene expression*.*

Methods

SNPs in the 5’ UTR of the *DEFB1 *gene (-20G>A (rs11362), -44C>G (rs1800972), and -52G>A (rs1799946)) were genotyped by polymerase chain reaction (PCR) followed by Sanger sequencing. The study group included periodontitis (n = 40), periodontitis with T2DM (n = 20), and periodontally and systemically healthy as controls (n = 40). *DEFB1* gene expression was determined by real-time PCR in the study group comprising periodontitis (n = 20), periodontitis with T2DM (n = 15), and healthy controls (n = 20). The effect of 5’ UTR polymorphisms on the expression was analyzed by statistical tools.

Results

Statistically significant higher prevalence of the variant AA genotype of rs11362 was observed in periodontitis (odds ratio (OR) = 3.64, 95% confidence Interval (CI) = 1.16-11.43, p = 0.04) and periodontitis with T2DM (OR = 5.14, 95% CI = 1.29-20.5, p = 0.03) in comparison with healthy controls. Moreover, there was a significant increase of the variant AA genotype of rs1799946 in periodontitis (OR = 3.88, 95% CI = 1.19-12.68, p = 0.04) compared to healthy controls. *DEFB1 *gene expression was downregulated in periodontitis and upregulated in periodontitis with T2DM patients when compared to healthy controls but was not statistically significant. No significant association was found for the effect of SNPs of the *DEFB1* gene on its expression.

Conclusion

From the SNP analysis, it can be inferred that the presence of SNPs at the 5’ UTR (rs11362 and rs1799946) in the *DEFB1 *gene may be an important predictive factor for periodontitis.

## Introduction

Periodontitis, a multifactorial disease, is characterized by microbially triggered and host-mediated inflammation that result in the destruction of tooth-supporting tissues, including the periodontal ligament and alveolar bone [1]. Each patient differs in their immunological responses to the presence of periodontopathogenic bacteria, which is predominantly influenced by a person's genetic makeup [2]. Diabetes plays a major risk factor for periodontitis with a bidirectional relationship between the two diseases [3]. Type 2 diabetes mellitus (T2DM) has inflammation key to its disease progression, so it is suggested that there is a strong link between periodontitis and T2DM [4]. Both periodontitis and T2DM have a hereditary component and hence genetic factors are considered to play a role in the susceptibility to the diseases [5].

Single-nucleotide polymorphism (SNP) is a common type of sequence variation, and in contrast to mutations that have been linked with Mendelian diseases, genetic polymorphisms are often not directly linked, but rather specific alleles are reported to be found more frequently in diseased individuals than in non-affected controls [6]. It is imperative to identify genetic factors that play an important role in the predisposition to various diseases. The effect of polymorphisms in genes for a particular disease may not be the same in different ethnic populations, and several gene polymorphisms have an overall contribution of risk to the disease susceptibility and severity [7]. SNPs can occur in protein-coding or non-coding regions (untranslated region (UTR), intronic region, and intergenic region). SNPs in coding regions can disrupt the structure and function of a protein. The UTR region is associated with the control of transcription and translation, and polymorphisms in UTR can result in deregulation of gene expression [8].

Antimicrobial peptides (AMPs) are constituents of innate immune responses and are produced by epithelial cells and other immune cells, such as neutrophils, against pathogen invasion. Defensins are short cationic AMPs possessing broad-spectrum antimicrobial activity against gram-positive and gram-negative bacteria, viruses, and fungi. It has been speculated that β-defensin 1 (*DEFB1* gene) is continuously expressed in the oral cavity and plays a role in preventing normal flora from becoming opportunistic [9]. It kills bacteria by targeting its membrane leading to osmotic imbalance. Genetic variations in the *DEFB1* gene could affect gene expression and increase the susceptibility to periodontitis by having reduced capability to attack the pathogens, hence leading to the disease. Several polymorphism studies in periodontitis including matrix metalloproteinase 3 [10], interleukin 16 (IL-16) [11], IL1α [12], and tumor necrosis factor α (TNFα) [13] have been carried out. Our study analyzed the effect of genetic variations in 5’ UTR (rs11362 (-20G>A), rs1800972 (-44C>G), and rs1799946 (-52G>A)) of the *DEFB1* gene on the susceptibility to periodontitis associated with T2DM in the south Indian population and their influence on gene expression.

## Materials and methods

Study population

The participants were recruited from the outpatient Department of Periodontics and Department of Oral Medicine & Radiology, Sree Balaji Dental College & Hospital, Chennai, Tamil Nadu, India. This study was approved by the Ethical Committee of Sree Balaji Dental College & Hospital (reference no.: SBDCH/IEC/12/2019/1 dated 18/12/2019). Written informed consent was obtained from the participants. Based on periodontal health and systemic conditions, the participants were divided into three groups: (i) periodontitis alone, (ii) periodontitis with T2DM, and (iii) periodontally and systemically healthy individuals as controls. The sample size was determined based on a prior report of a prevalence of 42.3% of periodontitis in the south Indian population [14]. With 43% incidence in the patient group and assuming 5% in controls with odds ratio (OR) of 1 and a power of 80%, a minimum of 19 cases and 19 control samples are needed [15]. The study population comprised individuals of South Indian origin. Individuals aged 30-75 participated in this study, including 40 periodontitis, 20 periodontitis with T2DM, and 40 periodontally and systemically healthy participants.

Clinical examination

Periodontitis was diagnosed according to the 2017 Classification of Periodontal and Peri-Implant Diseases and Conditions [16]. The subjects were diagnosed with periodontitis if they had bleeding on probing (BOP) ≥10% and interdental clinical attachment loss (CAL) detectable at ≥2 non-adjacent teeth with periodontal pocket depth (PPD) ≥4 mm. T2DM patients were diagnosed according to the American Diabetes Association’s 2020 guidelines [17]. Periodontitis with T2DM patients (glycatedhemoglobinA1c (HbA1c) ≥ 6.5%, fasting plasma glucose ≥ 126 mg/dL) were considered in this study. In the present study, HbA1c levels in periodontitis with T2DM patients were less than 7.5%. The following individuals were excluded from the study if they (i) were pregnant or lactating women; (ii) had received antibiotic treatment in the previous three months; (iii) were taking anti-inflammatory or immunosuppressive drugs; (iv) were diagnosed with systemic conditions, including human immunodeficiency virus infection, cardiovascular disorders, and renal disorders; and (v) were smokers.

Isolation of genomic DNA

Unstimulated saliva was collected from the participants according to Navazesh (1993) [18], and genomic DNA was isolated by a standard salting-out method. Saliva samples were centrifuged at 10000 rpm for 10 minutes. The supernatant was discarded, and then 1000 µl of lysis buffer (50 mM Tris-HCl at pH 8, 10 mM ethylenediaminetetraacetic acid, and 0.2% sodium dodecyl sulfate) was added to the pellet and incubated at 65°C for two hours. The samples were cooled to room temperature, and 5 M NaCl was added to each tube and vortexed. The samples were kept at 4°C for 10 minutes and then centrifuged at 10000 rpm for 10 minutes. An equal volume of isopropanol was added to the supernatant and centrifuged for five minutes. To the pellet, 70% ethanol was added and centrifuged for five minutes. The supernatant was discarded without dislodging the pellet. The DNA pellet was air-dried and dissolved in 100 µl of nuclease-free water and stored at -20°C for further use. The quality and quantity of DNA were checked using 1% agarose gel electrophoresis and the Quantus Fluorometer (Promega, USA), respectively.

Genotyping by Sanger sequencing

The polymorphisms (rs11362, rs1800972, and rs1799946) of 5’ UTR of the *DEFB1* gene were genotyped by polymerase chain reaction (PCR) using primer sequences and thermal cycling parameters as described previously in the literature [19], followed by the Sanger method of sequencing. Primer sequences covering three closely located 5’ UTR SNPs were used for amplification and sequencing and are given in Table 1. PCR was performed with 50-100 ng of DNA under the following conditions. After the initial denaturation at 95°C for 15 minutes, the reaction mixture was subjected to 35 cycles of 94°C for 30 seconds, annealing for 30 seconds at 67°C, and 72°C for 30 seconds, followed by the final extension at 72°C for five minutes. In a 1% agarose gel, samples migrated at the expected size of 261 bp, and PCR-amplified products were given for DNA sequencing.

**Table 1:** PCR primer sequences of the DEFB1 gene Citation: Wallace AM et al. (2006) [19]

DEFB1 gene	Primers
Forward primer	5'-GTGGCACCTCCCTTCAGTTCCG
Reverse primer	5'-CAGCCCTGGGGATGGGAAACTC

Gene expression analysis

Isolation of RNA from Saliva

The sample size for gene expression was arrived with nMaster software version 2.0 (Department of Biostatistics, USA) by applying a mean difference of 10, effect size 2.9, alpha error (%) 1, and power ((1-β)%) 95, and the minimum required sample size is 4. Saliva samples (20 periodontitis, 15 periodontitis with T2DM, and 20 healthy controls) stored in RNAlater were processed for RNA isolation. The samples were centrifuged at 10000 rpm, 4°C for 10 minutes. The supernatant was removed and to the pellet, and 1 ml of RNA iso Plus (Takara, USA) was added and vortexed for two minutes. The samples were incubated for five minutes at room temperature, and 200 µl of chloroform was added, vortexed, and centrifuged at maximum speed for 10 minutes. The aqueous phase was transferred to a fresh tube, and sodium acetate (~75 mM) was added and vortexed. Double the volume of ice-cold isopropanol was added and incubated at -20°C overnight. On the next day, the samples were centrifuged at 13,000 rpm for 10 minutes, and the supernatant was discarded. To the pellet, 1 ml of 70 % ethanol was added and vortexed. The samples were centrifuged at maximum speed for one minute, and the supernatant was discarded. The pellet was air-dried and 30 µl of RNase-free water was added to re-dissolve the RNA pellet.

*Gene Expression by Quantitative* *Real-Time Reverse Transcriptase PCR (qRT-PCR)*

Isolated RNA was quantified by the Quantus Fluorometer (Promega) and ~1 μg of RNA was used for cDNA synthesis. High-capacity cDNA conversion kit (Thermo Fisher Scientific Inc., USA) was used to reverse-transcribe RNA to cDNA according to the manufacturer’s protocol.

The KAPA SYBR GREEN qPCR master mix (Sigma-Aldrich, USA) was used to carry out the gene expression analysis in the Rotor-Gene Q (QIAGEN, Germany) instrument using the primer pairs as given in Table 2, with the following cycle conditions as given by the manufacturer: enzyme activation 95°C for three minutes, 40 cycles of denaturation 95°C for three seconds, and annealing 60°C for 30 seconds. The melt curve was set according to the instrument guidelines. Glyceraldehyde-3-phosphate dehydrogenase (GAPDH) gene was used as an internal control.

**Table 2:** Primers used for the gene expression analysis FP: forward primer, RP: reverse primer

Genes	Primer sequence	Length
DEFB1	FP: 5’ CTGAAATCCTGRGTGTTGCC (R=G/A) RP: 5’ GTAAAGATCGGGCAGGCAGA	189 bp
GAPDH	FP: 5’ CTGACTTCAACAGCGACACC RP:5’ TGCTGTAGCCAAATTCGTTGT	114 bp

Statistical analysis

Epi Info software version 7.0 (Centers for Disease Control and Prevention, Atlanta, Georgia) was used to perform statistical calculations for the polymorphism analysis. For both cases and controls, Hardy-Weinberg equilibrium (HWE) was determined for all the SNPs. The distribution of genotype and allele frequencies in the groups was compared using the Chi-square (χ2-test) goodness-of-fit test. OR with 95% confidence interval (CI) was calculated to find the risk associated with the genotypes. Statistical significance in the test was set at p < 0.05.

The expression of the *DEFB1* gene was studied using the SYBR green dye, and the relative amount of mRNA specific to the *DEFB1* gene was calculated using the 2^-ΔΔCT method [20] with the GAPDH gene for normalization. T-test was applied to check the p-value significance.

## Results

Genotyping of the *DEFB1* gene by PCR and sequencing

**Figure 1:**
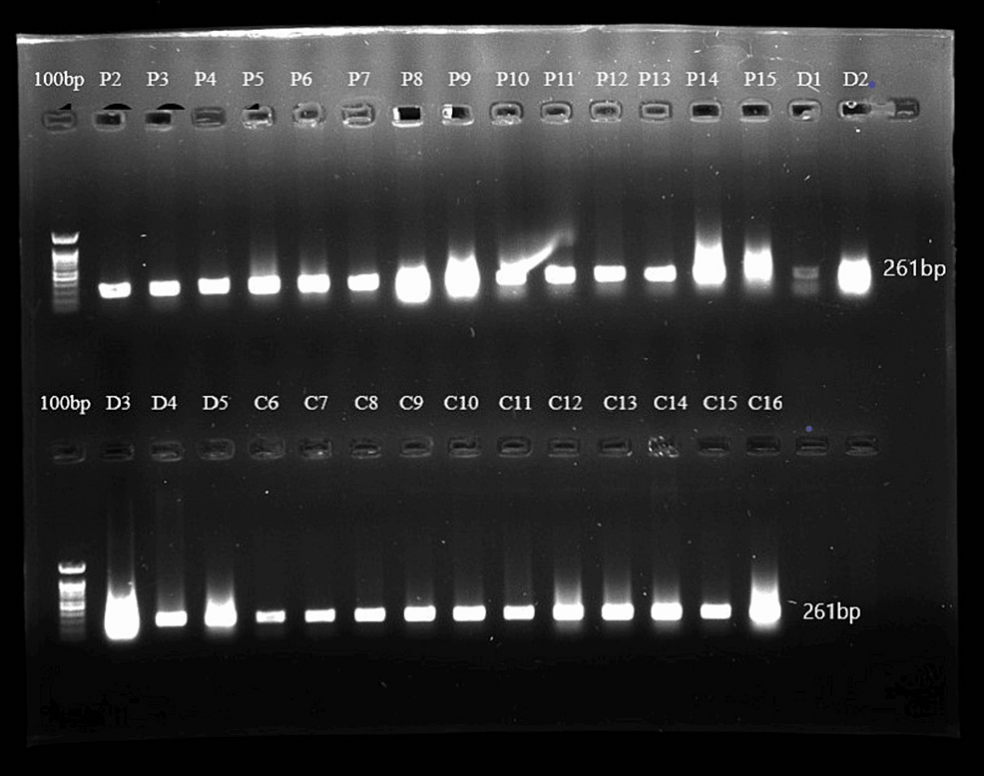
Representative image of the DEFB1 gene amplification by PCR. 100 bp marker, P2-P15: periodontitis patients, D1-D5: periodontitis with T2DM, C6-C16: healthy controls. PCR: polymerase chain reaction. The product size is 261 bp.

**Figure 2:**
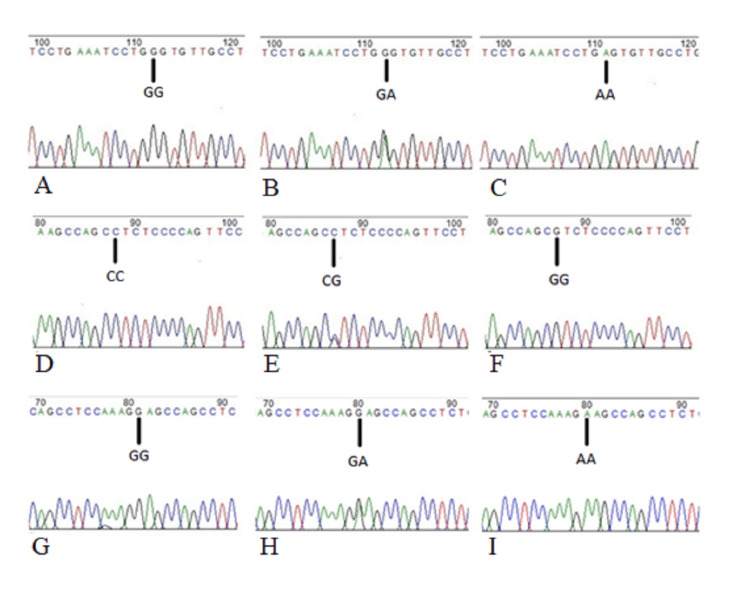
Representative image of Sanger sequencing rs11362 SNP: A) GG, B) GA, C) AA;  rs1800972 SNP: D) CC, E) CG, F) GG; rs1799946 SNP: G) GG, H) GA, I) AA

5’ UTR polymorphisms of the *DEFB1* gene (rs11362, rs1800972, and rs1799946) were studied to check if they had any association with susceptibility to periodontitis and also periodontitis with T2DM conditions. DNA isolated from saliva was amplified for the *DEFB1* gene, and the representative gel image of amplification is shown in Figure 1. SNP analysis by sequencing provided genotyping data, which were categorized into homozygous wild type, heterozygous, and homozygous variants, and the chromatogram is shown in Figure. 2.

The genotype and allele frequencies were calculated for both cases and controls and are shown in Tables 3-5. The genotype distributions of the *DEFB1* SNPs did not deviate from the HWE in controls (p-value > 0.05). For periodontitis with T2DM individuals, the genotype distributions did not deviate from the HWE (p-value > 0.05) except rs11362. Moreover, the genotype frequencies were consistent with the HWE for periodontitis patients for only rs1799946 SNP (p-value > 0.05). The variant AA genotype of rs11362 showed a significant increase in the periodontitis patients (OR = 3.64, 95% CI = 1.16-11.43, p = 0.04) and periodontitis with T2DM patients (OR = 5.14, 95% CI = 1.29-20.5, p = 0.03) when compared to the healthy controls. This indicates that the variant AA of rs11362 is associated with the increased risk of periodontitis and periodontitis with T2DM. The variant AA genotype of rs1799946 also showed a significant increase in the periodontitis patients (OR = 3.88, 95% CI = 1.19-12.68, p = 0.04) compared to the healthy controls, indicating that it is associated with the increased risk of periodontitis. rs1800972 SNP did not show any significant association with the disease conditions.

**Table 3:** Genotype frequency distribution for 5’ UTR DEFB1 gene polymorphisms T2DM: type 2 diabetes mellitus, UTR: untranslated region

Genotypes	Periodontitis (n = 40)	Periodontitis+T2DM (n = 20)	Healthy controls (n = 40)
rs11362			
GG	12 (30%)	5 (25%)	18 (45%)
GA	11(27.5%)	5 (25%)	15 (37.5%)
AA	17(42.5%)	10 (50%)	7 (17.5%)
rs1800972			
CC	25 (62.5%)	13 (65%)	22 (55%)
CG	9 (22.5%)	5 (25%)	13 (32.5%)
GG	6(15%)	2 (10%)	5 (12.5%)
rs1799946			
GG	10 (25%)	7 (35%)	17 (42.5%)
GA	14 (35%)	8 (40%)	16 (40%)
AA	16 (40%)	5 (25%)	7 (17.5%)

**Table 4:** Association analysis of DEFB1 gene polymorphisms with the disease and control groups CI: 95% confidence interval, OR: odds ratio, p: p value (*p < 0.05 = statistically significant), Ref.: reference

Genotypes	Periodontitis vs. Healthy		Periodontitis+T2DM vs. Healthy		Periodontitis+T2DM vs. Periodontitis	
	OR (CI)	p	OR (CI)	p	OR (CI)	p
rs11362						
GG	Ref.		Ref.		Ref.	
GA	1.1 (0.37-3.19)	1	1.2 (0.29-4.94)	1	1.09 (0.24-4.81)	1
AA	3.64 (1.16-11.43)	*0.04	5.14 (1.29-20.5)	*0.03	1.41 (0.38-5.19)	0.8
rs1800972						
CC	Ref.		Ref.		Ref.	
CG	0.6 (0.21-1.69)	0.48	0.65 (0.18-2.24)	0.7	1.06 (0.29-3.85)	1
GG	1.05 (0.28-3.94)	1	0.67 (0.11-4.0)	1	0.64 (0.11-3.6)	0.9
rs1799946						
GG	Ref.		Ref.		Ref.	
GA	1.48 (0.51-4.29)	0.64	1.21 (0.35-4.12)	1	0.81 (0.22-2.99)	1
AA	3.88 (1.19-12.68)	*0.04	1.73 (0.4-7.36)	0.7	0.44 (0.11-1.79)	0.4

**Table 5:** Allele frequency distribution for DEFB1 gene polymorphisms T2DM: type 2 diabetes mellitus

Allele distribution	Periodontitis	Periodontitis+T2DM	Healthy controls
rs11362			
G	0.44	0.37	0.64
A	0.56	0.63	0.36
rs1800972			
C	0.74	0.77	0.71
G	0.26	0.23	0.29
rs1799946			
G	0.42	0.55	0.62
A	0.58	0.45	0.38

*DEFB1 *gene expression analysis

**Figure 3:**
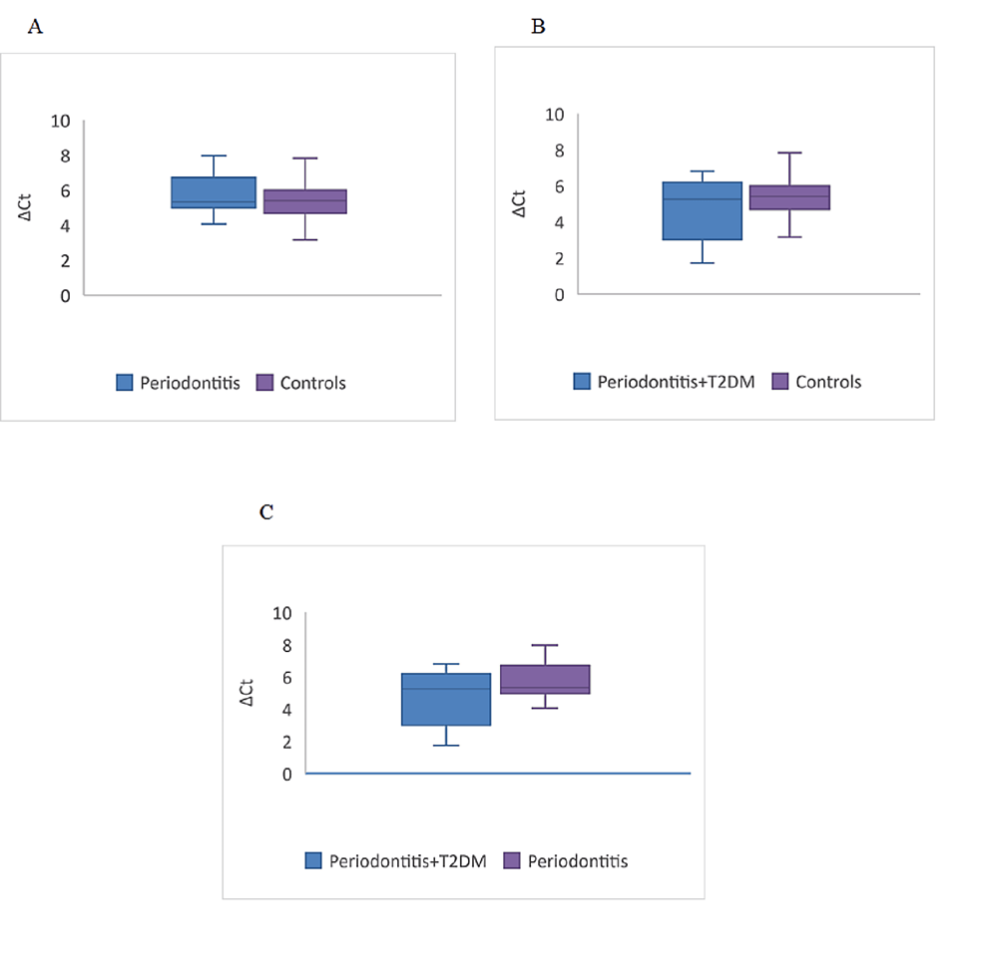
Relative expression of the DEFB1 gene between cases and controls The p-value was significant for the comparison between periodontitis+T2DM vs. periodontitis (p = 0.048).

**Figure 4:**
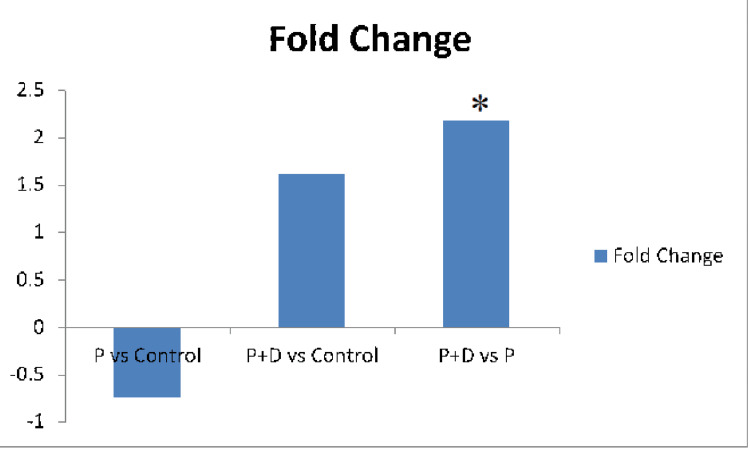
Fold change between cases and controls for the DEFB1 gene P: periodontitis patients, P+D: periodontitis with T2DM patients. *p-value (0.048) - fold change was significant for comparison between P+D vs. P.

Internal control gene *GAPDH *was used for data normalization. ∆Ct values and fold change between the groups are shown in Figure 3 and Figure 4, respectively. Fold changes between cases and controls are given in Table 6. The fold change was found to be 0.75, which is 25% downregulated when compared between periodontitis and healthy controls. When periodontitis with T2DM patients were compared with healthy controls, the fold increase was 1.61 times in periodontitis with T2DM patients. However, these relative quantifications of *DEFB1* gene expression showed no statistical significance between cases and controls. When periodontitis with T2DM patients was compared with periodontitis alone, the fold change was two times upregulated in periodontitis with the T2DM patient group. This comparison was found to be statistically significant (p = 0.048).

**Table 6:** Comparison of gene expression between cases and controls

Comparison	Fold change	Significance
Periodontitis vs. control	0.75 (25 % downregulated in periodontitis)	Not significant
Periodontitis with T2DM vs. control	1.61 times higher in periodontitis with T2DM patients	Not significant
Periodontitis with T2DM vs. periodontitis	2 times upregulated in periodontitis with T2DM patients	Significant (p = 0.048)

*DEFB1* gene expression analysis in association with the SNP study

To determine the effect of 5’ UTR SNPs of the *DEFB1* gene on its expression, the average ∆Ct values for each genotype were compared between cases and controls. Sequencing analysis carried out in this study revealed that the variant AA of rs11362 SNP is associated with periodontitis and periodontitis with T2DM, and the variant AA of rs1799946 is associated with periodontitis alone. Therefore, to analyze whether the variants of 5’ UTR rs11362 and rs1799946 SNPs had any influence on the gene expression, ∆Ct values of the variant genotypes were compared between disease and controls.

The results showed that the expression of the *DEFB1* gene was 59% downregulated (fold change = 0.41) for the AA variant genotype of rs11362 in periodontitis patients when compared with healthy controls, but it was not statistically significant (p = 0.07). No significant association was found for the effect of the rs11362 AA variant on the expression in periodontitis with T2DM patients. The statistical analysis of the effect of the rs1799946 AA variant on the gene expression indicated that the expression was 48% downregulated (fold change = 0.52) when compared to controls; however, the effect was not statistically significant (p = 0.130).

## Discussion

The study conducted in the Brazilian population revealed that the -20GG genotype of rs11362 was more frequent in periodontitis and periodontitis with T2DM patients [21]. In a study conducted in an Italian population, g.-20G>A (rs11362) and g.-44C>G (rs1800972) SNPs were found to have significant associations with periodontitis [22]. The study showed that the -20G/G genotype was more frequent in patients than in healthy controls and the -44C/C genotype was found to be more among healthy individuals than patients. The study conducted in the Japanese population revealed that the -44C/C (rs1800972) genotype was associated with severe chronic periodontitis [23]. In the present study, in contrast to the above studies, the variant AA genotype of rs11362 was found to be associated with an increased risk of periodontitis and periodontitis with T2DM. Moreover, the variant AA genotype of rs1799946 was found to be associated with periodontitis. No association was found for the rs1800972 SNP. The variants of rs11362 and rs1799946 of *DEFB1* 5’UTR polymorphisms might influence the susceptibility and progression of periodontitis.

The gene expression of β-defensins was high in non-inflamed oral tissues compared to inflamed samples [24,25]. In the present study, the expression of the *DEFB1* gene was downregulated in periodontitis and upregulated in periodontitis with T2DM patients compared to healthy controls but showed no statistical significance. However, when periodontitis with T2DM patients was compared with periodontitis alone, the expression was two times upregulated in the former group. This comparison was found to be statistically significant. The study showed that the variant AA of rs11362 and rs1799946 downregulated the *DEFB1* gene expression, but this effect was not statistically significant.

The DEFB1 protein links innate and adaptive immune systems through the chemotaxis of immature dendritic cells and memory T cells by interacting with the chemokine receptor CCR6 expressed on these cells [26]. Hence, the decreased expression of the *DEFB1* gene might affect immune responses against oral pathogens. Dissemination of periodontal pathogens into the systemic circulation can result in increased inflammatory responses and linked with systemic disorders, such as diabetes and cardiovascular disorders [27]. This study has identified polymorphisms in the *DEFB1 *gene to influence the development of periodontitis and periodontitis with T2DM and therefore could be considered a potential marker for identifying susceptible individuals. The peptides can be synthesized and used therapeutically in topical applications, mouthwash, or toothpaste, and this, in turn, can reduce the severity of the disease. Periodontitis has been associated with several systemic diseases, and identifying polymorphisms in periodontitis can help in altering treatment options for associated systemic illness. To understand the role of polymorphisms of components of innate immune defense, large-scale genetic variant analyses of coding sequence and regulatory regions are necessary. Different ethnic or geographic constitutions can have different genetic associations, so results from one study may not be similar to studies from other populations. The findings of the present study may have limitations with respect to the sample size. Also, the ethnic composition of the study population can influence the results. To get a more generalizable result, larger and more diverse samples are required. The effect of SNPs on the susceptibility of individuals to periodontitis is likely influenced by environmental factors or other gene variants due to potential interactions between genes and environmental factors. This study focused on the 5’ UTR variations of the *DEFB1* gene, and to draw a comprehensive inference, extensive analysis should be done integrating genetic and environmental factors.

## Conclusions

The results of the present study indicate that the variant of rs11362 of the *DEFB1 *gene was associated with periodontitis and periodontitis with T2DM. The variant of rs1799946 was associated with periodontitis alone. The variants did not have a significant effect on gene expression. Identification of genetic risk factors can help in developing new tools for early diagnosis, prevention, and better prognosis. Understanding the contribution of defensin in periodontitis can open new avenues of treatment, and personalized medications can be developed in the near future.
